# A mutant cotton *fatty acid desaturase 2-1d* allele causes protein mistargeting and altered seed oil composition

**DOI:** 10.1186/s12870-023-04160-8

**Published:** 2023-03-17

**Authors:** Jay Shockey, Matthew K. Gilbert, Gregory N. Thyssen

**Affiliations:** United States Department of Agriculture, Agricultural Research Service, Southern Regional Research Center, 1100 Allen Toussaint Blvd, New Orleans, LA 70124 USA

**Keywords:** Cottonseed, Fatty acid desaturase, Oleic acid, Linoleic acid, *Arabidopsis*, Transposable element, Retrotransposon, Ty1/*copia*

## Abstract

**Background:**

Cotton (*Gossypium* sp.) has been cultivated for centuries for its spinnable fibers, but its seed oil also possesses untapped economic potential if, improvements could be made to its oleic acid content.

**Results:**

Previous studies, including those from our laboratory, identified pima accessions containing approximately doubled levels of seed oil oleic acid, compared to standard upland cottonseed oil. Here, the molecular properties of a fatty acid desaturase encoded by a mutant allele identified by genome sequencing in an earlier analysis were analyzed. The mutant sequence is predicted to encode a *C*-terminally truncated protein lacking nine residues, including a predicted endoplasmic reticulum membrane retrieval motif. We determined that the mutation was caused by a relatively recent movement of a Ty1/*copia* type retrotransposon that is not found associated with this desaturase gene in other sequenced cotton genomes. The mutant desaturase, along with its repaired isozyme and the wild-type A-subgenome homoeologous protein were expressed in transgenic yeast and stably transformed *Arabidopsis* plants. All full-length enzymes efficiently converted oleic acid to linoleic acid. The mutant desaturase protein produced only trace amounts of linoleic acid, and only when strongly overexpressed in yeast cells, indicating that the missing *C*-terminal amino acid residues are not strictly required for enzyme activity, yet are necessary for proper subcellular targeting to the endoplasmic reticulum membrane.

**Conclusion:**

These results provide the biochemical underpinning that links a genetic lesion present in a limited group of South American pima cotton accessions and their rare seed oil oleic acid traits. Markers developed to the mutant desaturase allele are currently being used in breeding programs designed to introduce this trait into agronomic upland cotton varieties.

**Supplementary Information:**

The online version contains supplementary material available at 10.1186/s12870-023-04160-8.

## Background

Cotton (*Gossypium* sp.) is a biologically diverse and economically vital genus. Previous studies have identified eight monophyletic groups, designated by single letter genome groups “A” through “G” and “K,” comprising approximately 45 diploid species [1]. Additional genetic complexity occurred via an ancient hybridization about 1–2 million years ago, giving rise to new allopolyploid descendants [2], including the now commonly known upland cotton (*Gossypium hirsutum*, AD1) and pima cotton (*Gossypium barbadense*, AD2). These two allotetraploids, along with two African-Asian A diploids (*Gossypium herbaceum* (A1) and *Gossypium arboreum* (A2)) were subsequently domesticated and exploited for their production of cotton fiber, which now makes up the dominant global source of spinnable natural fibers for production of clothing and hundreds of other uses [3].

The other major co-product from fiber production is cottonseed, which on a weight basis, is more substantial than fiber. The seed/fiber ratio is 1.5–1.7 lbs of seed/pound of fiber, and cottonseed oil accounts for ~ 15–25% of the value of the crop. Cottonseed oil makes up a significant portion of the global vegetable oil market, which produces a total annual value of > $US240 billion [4]. In the past, cottonseed oils were used extensively in a variety of food applications, including frying oils. However, in recent years cottonseed oils have lost market share due to suboptimal fatty acid ratios in traditional cottonseed oil [5]. Historically, partial hydrogenation of plant oils altered these ratios to achieve the chemical and physical properties necessary for manufacturing of various semi-solid fat products, such as margarines and shortenings. But decades of research reached a clear consensus that hydrogenated oils contained high amounts of ‘trans-fats’, consumption of which contributed to the risk of heart disease and other negative health outcomes [6]. Some estimates have linked ~ 500,000 global excess deaths annually to consumption of trans-fats [7], and the Centers for Disease Control conservatively attributed approximately 20,000 excess deaths in the United States to trans-fats in 2010 alone, equivalent to the number of opioid overdose deaths in that year [8]. In 2018, the deadline imposed by the Food and Drug Administration [9] took effect, declaring that all partially hydrogenated oils would lose the important Generally Regarded As Safe (GRAS) status, and effectively dictated the elimination of trans-fats from all manufactured foods.

The naturally-occurring monounsaturated fatty acid oleic acid is the factor that must be manipulated to achieve the desired changes in metabolic compatibility [10] while maintaining (or increasing) oxidative stability of modified vegetable oils. Standard cottonseed oil contains only 14–17% oleic acid, and approximately three times (52–55%) as much polyunsaturated linoleic acid (C18:2) [5, 11]. Previous studies [11, 12] produced new cotton germplasm with large increases in seed oil oleic acid content but did so via genetic modification. More recently, Chen et al. [13] created cotton lines containing > 70% oleic acid through gene editing (‘CRISPR’). Societal and regulatory agency acceptance of products created with CRISPR technology around the world is still an ongoing process. The uncertainty in this regard, combined with the public wariness associated with genetically modified organisms and the dictates of the trans fats ban, has greatly increased the need to identify cotton accessions that naturally produce heart-healthy seed oils enriched in oleic acid.

Recently, we identified pima cotton accessions that produce twice the level of seed oil oleic acid found in standard upland cottonseed and identified a gene allele that accounted for most of the observed trait in accession Gb713 [14]; this trait was bred into agronomic fiber varieties of upland cotton and released to the public [15]. The causative alteration to the allele in question, called *fatty acid desaturase 2-1d*, was originally ascribed to a small DNA insertion near the 3’ end of the coding sequence of this gene. Here, we characterize this allele more fully, including identification of the mutagenizing insertion as being a full-length Ty1/*copia*-type transposable element that is present in three geographically clustered accessions that contain the seed oleate trait, including Gb713. We also have extended the tentative biochemical studies of the desaturase enzyme produced from the mutant allele to include expression studies in transgenic microbes and plants, to better understand the exact nature of the link between the transposon-induced mutation and the seed oil chemical trait.

## Results

### Transposable element identification and molecular characterization

We originally found Gb713 as part of a survey of > 1000 cotton accessions present in the U.S. National Cotton Germplasm Collection (NCGC), which was undertaken to identify cottonseeds with altered fatty acid composition, with a particular focus on elevated oleic acid content. In this survey, we also identified two other pima cotton (*G. barbadense*) accessions, Gb331 and Gb841, that contained similarly large increases in oleic acid content, paired with proportional decreases in linoleic acid. Linoleic acid is produced from phosphatidylcholine-bound oleic acid by fatty acid desaturases (FADs), in particular *FAD2* [16]. We identified a family of eight *FAD2* genes in Gb713 [14]. The sequences of seven of the candidate *FAD2* genes aligned well with known FAD2s from other cotton accessions and higher plants. Although initial contig assemblies did not provide thorough coverage of the entire *FAD2-1D* gene, the sequenced genome of Gb713 revealed an apparent mutation near the 3’ end of the coding sequence, caused by insertion from an unknown source. This mutation truncated the *FAD2-1D* protein coding sequence by introduction of a premature stop codon and altered the coding residues adjacent to the premature stop [14]. Simultaneously, Sturtevant et al. [17] characterized the *FAD2-1D* locus in Gb331 and found that it was altered by an identical insertion.

**Fig. 1:**
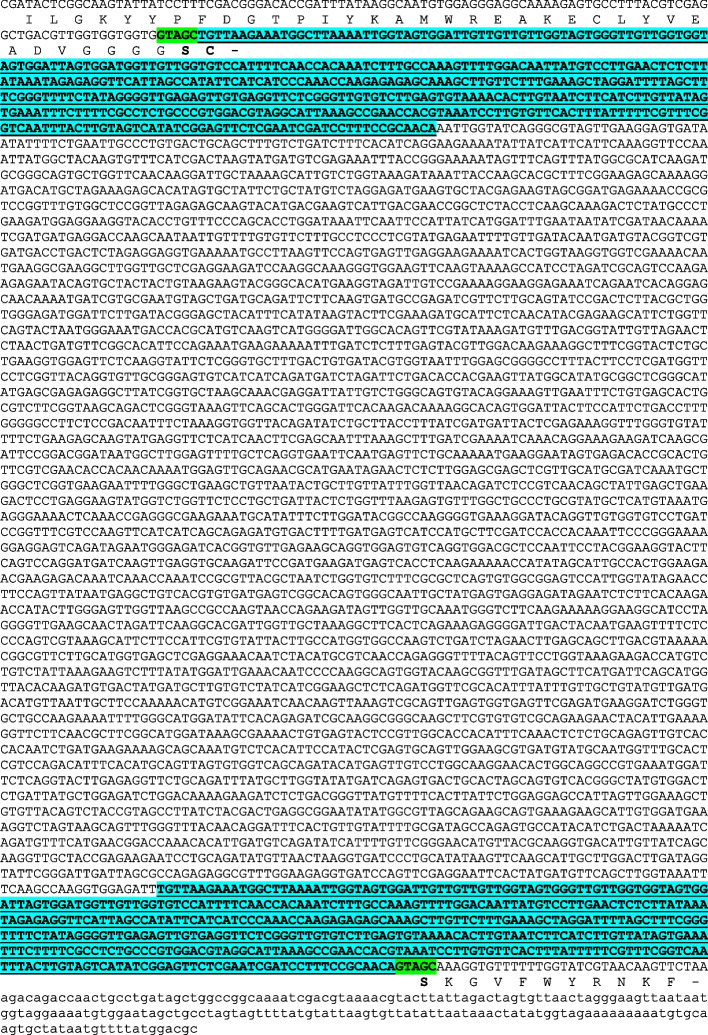
Gb713 *fad2-1d* Ty1/*copia* transposable element sequence. *FAD2-1D* protein-coding sequence capitalized and translated, 3’ untranslated sequence in lower case font. Duplicated long terminal repeats (LTRs) in bold, underlined, and shaded in blue. Target site duplications (TSD) in bold and boxed in green, upstream TSD created aberrant -Ser-Cys-* protein coding terminus; naturally-occurring *C*-terminal sequence located downstream of second TSD

As we analyzed the genomic DNA sequence located progressively downstream from the end of the Gb713 *FAD2-1D* gene, we realized that the inserted DNA was much longer than originally thought. Genome-walking and additional sequencing revealed that the inserted sequence bore the hallmarks of a Ty1/*copia* type retrotransposon (Fig. 1). This particular insertion is not present in the reference genome sequences for the commonly studied *Gossypium barbadense* cv. 3–79 or *G. hirsutum* cv. TM-1 [18]. The *FAD2-1D* gene is annotated as GbM_D13G2655 in the 'Pima90' HEAU v1.0 *G. barbadense* reference genome and as GB_D13G2499 in the ZJU v1.1 assembly and is located at Gbar_D13:58,245,090–58,241,939 in the HAU v2.0 assembly, though not annotated. The closest ortholog of the interrupted *FAD2-1D* gene in the reference *G. hirsutum* assembly, Ghir_D13G023320 (HAU v1.1) also does not contain this transposon insertion event [18]. Interestingly, no copies of this 5,098-bp Copia LTR transposon (GOBAR_AA36262, GenBank: PPR84446) are present anywhere in the available reference genomes of the diploid *Gossypium* species, *G. raimondii* and *G. arboreum*, that are considered the progenitors of both commercially relevant tetraploids [18–20]. There are 284 copies of this transposon in *G. barbadense* and 124 in *G. hirsutum*, and only two of these insertions are at orthologous locations (Gbar_D11:54,673,555–54,698,637 = Ghir_D11:54,683,552–54,688,647; Gbar_D13:577,491–602,577 = Ghir_D13:587,481–592,577) (Supplemental Table S1) [21] suggesting recent activity of this transposon in both species. The perfect identity of the 459-bp LTRs and complete coding sequences of the transposon in the *FAD2-1D* gene in Gb713 further suggest that this element is autonomous, and that the insertion event was fairly recent, although prior to the breeding that differentiated the Gb713, Gb331, and Gb841 varieties.

**Fig. 2:**
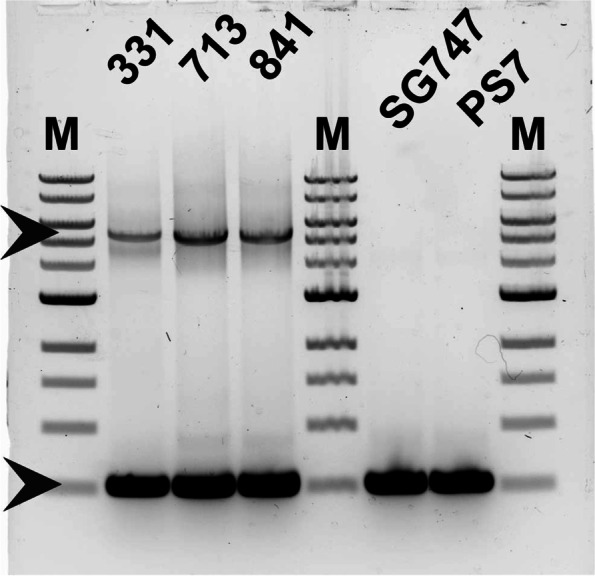
Visualization of ethidium bromide-stained agarose gel, displaying separation of ~ 5.44 kbp LTR retrotransposon-containing *fad2-1d* (upper arrow) and ~ 500 bp *FAD2-1A* (lower arrow) PCR amplicons from 3’ regions of high-oleate pima cotton accessions Gb331, Gb713, and Gb841. Control reactions from standard ‘normal-oleate’ varieties of *G. hirsutum* (SG747) and *G. barbadense* (Pima-S7, PS7) were included, note the lack of large PCR product in the controls. M denotes the 1 kb molecular weight marker (New England Biolabs)

To assess the similarities in this genomic region between the three high-oleate *G. barbadense* accessions, genomic DNA was also isolated from Gb331 and Gb841. Degenerate oligonucleotide primers that bind.near the respective 3’ ends of the protein coding sequences and in the untranslated 3’ flanking regions of both *FAD2-1D* and *FAD2-1A* (the A-subgenome homoeolog of *FAD2-1D*) from other publicly available cotton genome sequences [22] were used to perform long-distance PCR from all three high-oleate accession DNA samples, and two normal oleate accessions (*G. hirsutum* SG747 and *G. barbadense* PimaS7). Two bands, approximately 500 and 5500 bp in size respectively, were identified, with the smaller band present in all five samples and the larger band present only in the three high-oleate *G. barbadense* samples (Fig. 2). Cloning and sequencing of multiple individual copies of each of the PCR products shown in Fig. 2 revealed that all three high oleate accession derived ~ 500 bp bands represented wild-type *FAD2-1A*, while all three large bands were made up entirely of mutant *fad2-1d*, interrupted by the Ty1/*copia* retrotransposon described in Fig. 1. These data confirmed a shared causal link between the *fad2-1d* allele sequences and the high-oleate seed oil phenotypes observed in Gb713, Gb331, and Gb841. The annotations associated with these three accessions in the NCGC indicate that all three were identified in Peru, which suggest either a shared common ancestor or common breeding parent and is consistent with the location and close sequence identity of the three retrotransposons associated with the *fad2-1d* alleles.

### Lipid analysis of transgenic *Arabidopsis* seeds

To exploit the genetic resources inherent to the high-oleate trait present in Gb713, it is important to determine the nature of the genetic and biochemical changes caused by the *fad2-1d* mutation. Our preliminary study [14] showed that transient expression of different forms of Gb713 *FAD2-1A* and *fad2-1d* in *fad2-1* mutant *Arabidopsis* leaves [23] resulted in altered ratios of C18:1/C18:2, suggestive of different levels of FAD enzyme activity. Here, assessment of gene function was expanded to include a larger set of FAD2 constructs, containing different combinations of protein coding sequences and 3’ flanking regions. In addition to the alteration and deletion of C-terminal amino acid residues, the Gb713 *fad2-1d* allele also contains LTR retrotransposon sequence where its natural 3’ flanking sequence would reside, which may affect enzyme activity indirectly by altering mRNA transcript abundance or stability.

Each was built starting from plasmid E688, and empty vector negative control that contains only the V2 viral silencing suppressor gene [24]. All *FAD2* cassettes are driven by the *CVMV* promoter. ORFs are appended either directly to 3’ flanking sequences from GB-0713 *FAD2* genes or to the *ocs* terminator (*)-direct appendage of ORF to ocs terminator. _T_-truncated. _R_-repaired.

Table 1 lists the content of the binary plasmid DNA constructs that were constitutively overexpressed in organs (including seeds) of stably transformed *fad2-1* mutant *Arabidopsis* plants. The negative control contained only the *V2* viral silencing suppressor protein gene, which was included to attempt to suppress transgene silencing [24]. The other constructs also contained *V2*. Arabidopsis *FAD2*, flanked by the *CVMV* promoter and *ocs* terminator, was used as a positive control. All other constructs also contained the *CVMV* promoter. Some were paired with the *ocs* terminator, or the 3’ flanking regions from Gb713 *FAD2-1A* or *fad2-1d* in other cases, as discussed in the preceding paragraph. Selection of transgenic T_1_ plants on basta herbicide-treated soil was followed by gas chromatography (GC) quantification of total seed lipid fatty acid composition.

**Table 1:** Listing of FAD2 binary plasmid used in these studies

Plasmid stock #	Open reading frame	3’ flanking sequence
E689	Gb713 FAD2-1A	Gb713 FAD2-1A
E690	Gb713 FAD2-1D truncated	Gb713 FAD2-1D
E691	Gb713 FAD2-1D truncated	ocs terminator
E692	Gb713 FAD2-1D repaired	ocs terminator
E693	AtFAD2	ocs terminator
E694	Gb713 FAD2-1A	Gb713 FAD2-1D
E695 E860	Gb713 FAD2-1D truncated Gb713 FAD2-1D repaired	Gb713 FAD2-1A Gb713 FAD2-1D

**Fig. 3:**
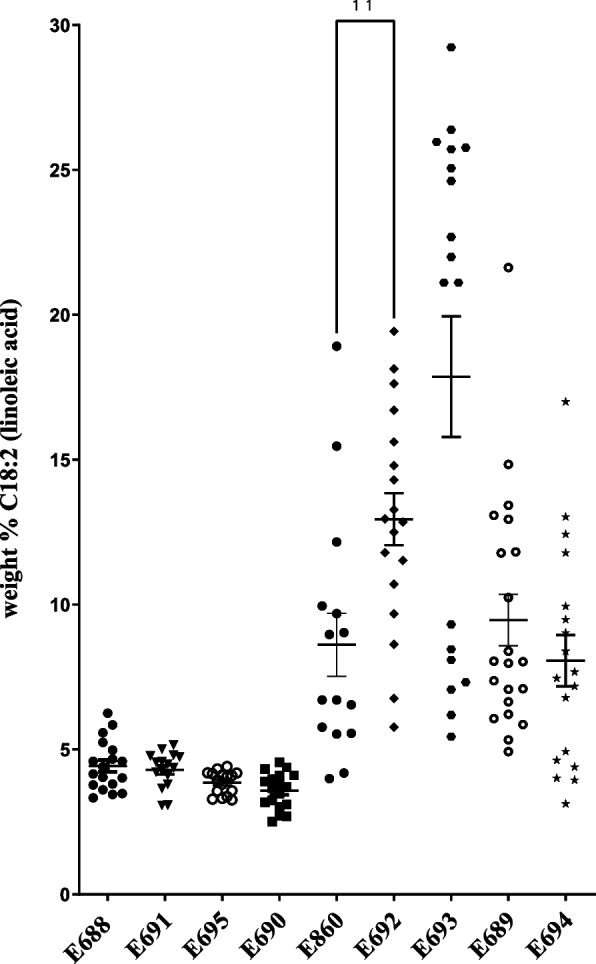
Total seed linoleic acid fatty acid methyl ester quantification from transgenic *fad2-1* mutant *Arabidopsis* seeds expressing various forms of Gb713 FAD2-1A and FAD2-1D fatty acid desaturases. Each data point represents the gas chromatographic value for segregating T_2_ seed samples from an independent transgenic event. Negative control empty vector (E688) and AtFAD2 positive controls (E692) are included. See Table 1 for specifics of each construct design. Repaired *FAD2-1D*, flanked by the *ocs* terminator (E692) produced significantly higher average amounts of C18:2 product compared to *FAD2-1D* fused to the LTR retrotransposon-derived 3’ flanking sequence (construct E860)

These data are summarized in Fig. 3, displayed as scatter plots, with each data point representing the weight percent of C18:2 linoleic acid, the product of the FAD2 reaction. As expected, seeds from the negative control averaged approximately 4.4% ± 0.9% C18:2 fatty acids, negligibly higher than the published 3.2% value [23]. Most of the positive control AtFAD2-expressing lines (E693, Table 1) restored linoleic acid production to near-normal levels for *Arabidopsis* seeds, with an overall average of ~ 18%, and several individual lines producing 20–30% C18:2. More importantly, all three lines expressing the predicted mutant form of Gb713 fad2-1d (E690, E691, and E695, Table 1) were statistically indistinguishable from the negative controls, with average linoleic acid levels between 3.6%-4.3% (Fig. 3). However, all four constructs expressing either the native FAD2-1A (E689, E694, Table 1) or repaired FAD2-1D enzymes (E692, E860, Table 1) produced appreciable amounts of linoleic acid relative to the negative controls.

### Lipid analysis of plant FAD-expressing yeast cells

**Fig. 4:**
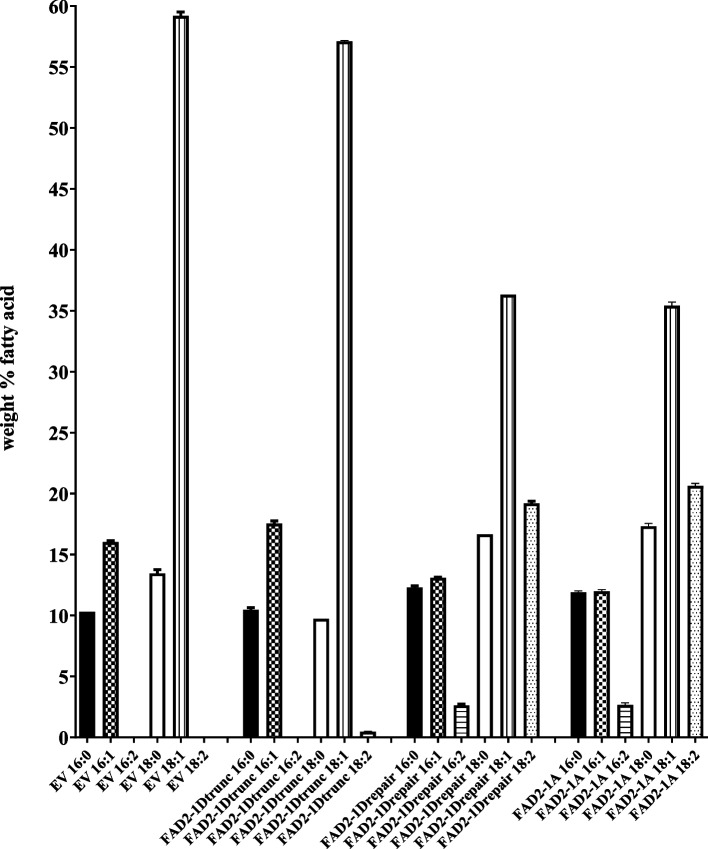
Total cellular lipid composition of baker’s yeast (*S. cerevisiae*) engineered to overexpress Gb713 FAD2-1A, and mutant or repaired FAD2-1D fatty acid desaturases. The values shown are the average of three replicates, error bars represent the standard deviations

FAD2-1A, mutant fad2-1d, and ‘repaired’ FAD2-1D were also studied by expression in baker’s yeast (*S. cerevisiae*). As shown in Fig. 4, GC quantification of total cellular lipids of the empty vector negative control strain revealed a typical profile dominated by the four common yeast fatty acids: C16:0, C16:1Δ9, C18:0, and C18:1Δ9 (oleic acid). Consistent with other recent plant FAD2 yeast expression studies [25, 26], yeast strains expressing repaired FAD21D and FAD21A produced 18.9% and 20.3% C18:2, with proportionate decreases in C18:1Δ9, in accordance with the substrate-product relationship between C18:1Δ9 and C18:2 (Fig. 4). These two enzymes each also produced ~ 2.5–2.8% 16:2, a polyunsaturated C16 secondary FAD2 product derived from the large pool of 16:1Δ9 in yeast. Mutant fad2-1d showed considerably less activity but did produce a very small amount of C18:2 (~ 0.5%, Fig. 4, Supplementary Fig. 1).

### FAD isozyme-specific subcellular targeting

**Fig. 5:**
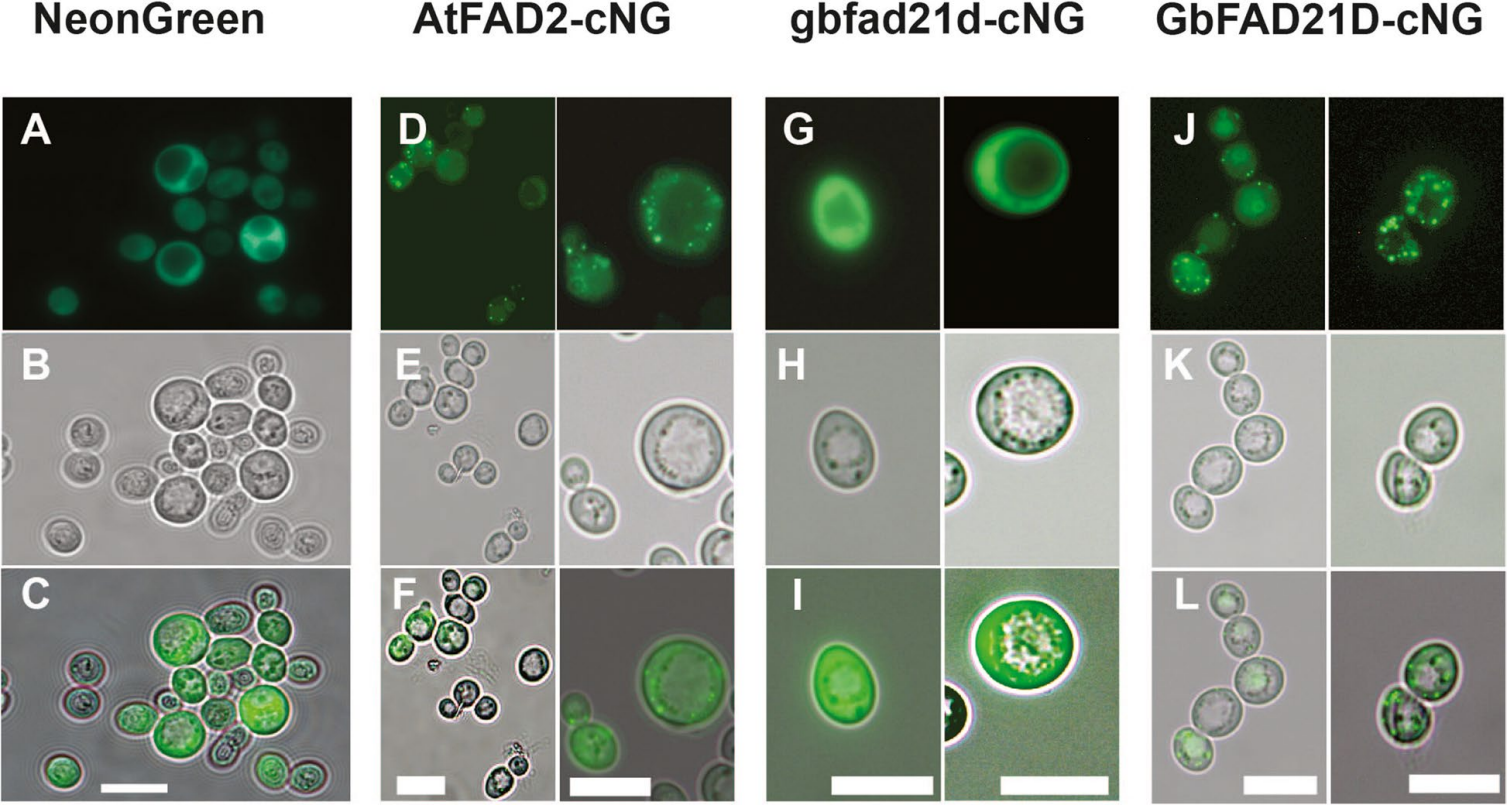
Light microscopy analysis of cellular fluorescence produced by baker’s yeast cells expressing plant FAD2/NeonGreen protein fusions. Construct carrying NeonGreen fluorescent protein alone is included as a control (A). Other constructs contain NeonGreen appended near the respective *C*-termini of AtFAD2 (AtFAD2-cNeonGreen, D), mutant fad2-1d (fad2-1d-cNeonGreen, G), and repaired FAD2-1D (FAD2-1D-cNeonGreen, J). Bright field images and merged images bright field and fluorescing cells are shown in panels B, E, H, and K, and C, F, I, and L, for NeonGreen alone, AtFAD2-cNeonGreen, fad2-1d-cNeonGreen, and FAD2-1D-cNeonGreen, respectively. The scale bar included in all panels = 10 μm

Cotton and *Arabidopsis FAD2* fusion protein genes, modified to contain the NeonGreen fluorescent protein coding sequence near the respective *C*-termini (Supplementary Table 2) were expressed in *S. cerevisiae* to investigate the subcellular destination of each fusion protein (Fig. 5). All constructs tested fluoresced brightly when the yeast cells were cultured in galactose and raffinose, which induce the *GAL10* promoter in the pESC-His vector used for cloning. Native NeonGreen was targeted to the yeast cytosol as expected (Fig. 5A), and the AtFAD2-cNeonGreen construct was primarily targeted to less intensely fluorescing reticular structures and more intensely fluorescing punctate structures that likely represent condensed regions of ER membrane (Fig. 5C). The fluorescence pattern of the mutant fad2-1d/NeonGreen fusion protein (Fig. 5E) resembled that of the cytosolic NeonGreen control construct, while repaired FAD2-1D (Fig. 5G) displayed the reticular/punctate pattern observed for the AtFAD2-cNeonGreen positive control.

## Discussion

Global demand for vegetable oils is growing at a rapid pace: increases in consumption have outpaced population growth for decades and represents ~ 35% of caloric intake in some western countries [27]. Though produced at significantly lower levels than other major oilseeds such as soybean or canola, cottonseed oil offers the strategic advantage of being the major co-product of cotton fiber processing, thus offering a degree of flexibility regarding which types of breeding and engineering might be useful in future cotton germplasm. The recognition of the human health risks posed by trans-fats has focused much of the projected future growth of vegetable oil markets, on the development of heart-healthy oleic acid-enriched oils, an area of cottonseed research that has borne productive results in preliminary studies [11, 12, 15]. However, full exploitation of the available cotton gene alleles that contribute to substantive changes in seed oil oleic acid content are best achieved through complete understanding of the exact nature of the biochemical and genetic changes conferred by these alleles.

Expanding upon an earlier study [14], we found that the mutant *fad2-1d* allele in three Peruvian pima cotton accessions was caused by the evolutionarily recent integration of a Ty1/*copia* type retrotransposon (Figs. 1, 2) near the 3’ end of the protein coding sequence. The possible biochemical effects of this insertion were two-fold. The first predicted change was the alteration of enzyme activity or subcellular targeting due to the alteration/loss of *C*-terminal amino acid residues, including two non-conservative changes and a premature stop codon, causing the loss of the nine extreme *C*-terminal residues (including those necessary for ER membrane retrieval) [28]. Secondly, the retrotransposon insertion created an entirely new 3’ flanking region downstream of the *fad2-1d* coding sequence, and it seemed possible these changes could alter mRNA transcript production and/or stability and thus the steady-state enzyme activity level. The direct role of the LTR retrotransposon on FAD2-1D-catalyzed production of linoleic acid due to truncation of the 3’ end of the protein coding sequence was clear; all plant and yeast expression lines containing the mutated FAD protein coding sequence produced little to no C18:2 product (Figs. 3, 4, Supplementary Fig. 1). Yeast is a very useful tool for genetic and biochemical studies of many types of plant genes and enzymes, but it is especially so for plant desaturases. This system produces all the necessary cofactors for higher-order fatty acid desaturation but does not naturally produce any polyunsaturated fatty acids on its own [28]. The yeast strain expressing mutant *fad2-1d* (Fig. 4) revealed one additional insight: the presence of ~ 0.5% C18:2. The large reduction of C18:2 production by mutant fad2-1d compared to repaired FAD2-1D (0.5% vs. 18.9% of total fatty acids) showed that the alteration in the protein coding sequence near the *C*-terminus had a clear negative effect on linoleic acid production. But the maintenance of residual levels of fad2-1d enzyme activity, made possible by the sensitive detection limits available in yeast, do strongly suggest that the lost and altered *C*-terminal residues in this allele are not strictly required for FAD2 enzyme catalysis, as was the case for an only slightly longer deletion found in the related desaturase AtFAD6 [29]. Instead, it seems very likely that the loss of the ER retrieval motif near the extreme *C*-terminus of fad2-1d and other plant FAD2s [28] leads to protein mistargeting. The altered subcellular targeting of a fad2-1d/fluorescent protein fusion, relative to AtFAD2- and repaired FAD2-1D fusions also supports these findings (Fig. 5).

The potential indirect effect of the retrotransposon on linoleic acid biosynthesis via altered transcript production or stability was less obvious. Repaired *FAD2-1D*, flanked by the strong *ocs* terminator (E692, Table 1, Fig. 3) produced significantly higher average amounts of C18:2 product (students’ T-test, *P* = 0.004) in transgenic *Arabidopsis* seeds compared to construct E860, which contains repaired *FAD2-1D* fused to the LTR retrotransposon-derived 3’ flanking sequence. However, the average transgenic seed performance of *FAD2-1A* fused to its own 3’ flanking region (E689, Table 1, Fig. 3) was statistically indistinguishable from *FAD2-1A* fused to the LTR retrotransposon-derived 3’ flanking from Gb713 *fad2-1d* (E694) (Fig. 3, students T-test *P* = 0.2756). Sturtevant et al. [17] observed low, but relatively unchanged, transcript levels for both *FAD2-1A* and *FAD2-1D* in high-oleate accession Gb331, compared to two other normal-oleate pima accessions, which also argues against the altered 3’ flanking region having a significant negative effect on *FAD2-1D* transcription in any of the three high-oleate Peruvian accessions, including Gb713.

## Conclusions

In this study we sought to gain a better understanding of the cause of the mutation in the *FAD2-1D* gene of a previously identified elevated-oleic acid accession of pima cotton, and the molecular properties conveyed to the mutant desaturase enzyme by this mutation. Rather than a relatively small DNA insertion as first thought, genome-walking and DNA sequencing revealed that the mutation was caused by the movement of a Ty1/*copia*-type retrotransposon. The mutation alters the 3’ end of the protein-coding sequence, altering two amino acid residues and deleting nine, resulting in a premature stop codon. Though none of the lost residues appear to be essential for enzyme activity, expression of the mutant form of fad2-1d in transgenic yeast and plants resulted in little to no linoleic acid production, while restoration of the proper *C*-terminal residues bestowed C18:2 production levels comparable to the native FAD2-1A homoeologous enzyme. Collectively these data confirm the importance of the lost *C*-terminal ER retrieval motif lost due to the *fad2-1d* mutation. This motif is typically found in most plant FAD2 enzymes and many other ER-resident integral membrane enzymes involved in lipid biosynthesis. These results also reaffirm the importance of FAD2 type desaturases as determinants of seed oil fatty acid composition, including cottonseed oil.

## Methods

### Plasmid design and yeast transformation

DNA sequences encoding fluorescent protein fusions were synthesized by Integrated DNA Technologies (Supplementary Table 1). This list includes the NeonGreen [30] open reading frame alone, or in open reading frames containing either *Arabidopsis FAD2* [23] or the *C*-terminally truncated *fad2-1d* allele found in Gb713. The NeonGreen coding sequence was inserted immediately downstream of the last transmembrane spanning domain, near the respective *C*-termini, and is preceded by an eight-residue glycine-serine linker sequence to allow for flexibility and proper protein folding. The full sequences and detailed descriptions of the synthetic sequences are shown in Supplementary Table 1. Primers pairs containing 5’ *Not*I and 3’*Pac*I recognition sequences were used to PCR amplify the synthetic genes, followed by restriction digestion, and cloning into the respective sites in the galactose-inducible yeast expression plasmid pESC-His (Agilent Technologies, Santa Clara, CA, USA).

### LTR-retrotransposon analysis

Sequencing of the large insertion in the FAD2-1D gene in the high-oleic cotton varieties was accomplished by Sanger sequencing and primer walking. Homology of the LTRs and identification of the 5-bp target site duplication were observed by alignment of the sequence to itself with LALIGN software [31]. The sequence of the 5098-bp insertion was used as a query for BLAST comparison to available sequences at NCBI and in the *G. barbadense, G. hirsutum, G. raimondii*, and *G. arboretum* reference genomes available at CottonGen [18, 32]. The positions of the full-length copies of the novel retrotransposon, which has high homology to GOBAR_AA36262 (GenBank: PPR84446), were used to extract reference sequences of highly homologous full-length retrotransposons from the reference genomes, with and without 10-kbp of flanking sequence. These sequences were aligned with MAFFT [33]. The alignment that included flanking sequences was used to identify ancestral insertions that are present in both tetraploid reference genomes. Distinction between *copia* and *gypsy* type LTR-transposons was conducted by analysis of the order of coding sequences in the open reading frame [34].

### Yeast cell fluorescent protein microscopy

Plasmids containing C-terminally NeonGreen-appended plant FAD2 (or NeonGreen alone) were transformed into yeast strain MMY011α as described previously [35] and colonies selected on solid media lacking either leucine or histidine, depending on the plasmid used for cloning. Colonies were grown in liquid media containing dextrose, then back-diluted into equivalent media containing raffinose and galactose to induce gene expression. After 24 h, cells from early- to mid-log phase cultures were re-suspended in 500 mM sorbitol and imaged using a 100X/1.25 oil immersion lens on an Eclipse E600 compound light microscope (Nikon Instruments Inc, Tokyo, Japan) using an Imaging Source model #77,020,507 digital camera (Imaging Source, LLC, Charlotte, NC, USA). GFP fluorescence was obtained using a Nikon Intensilight Illuminator C-HGFI with a Nikon 450-490EX/500-550EM filter. Images were acquired using NIS-Elements D Software (Nikon Instruments, Tokyo, Japan).

### Plant growth and transformation

*Arabidopsis* seeds (*fad2-1* mutant) [16] were sown on Sungro Mix 4 (Sun Gro Horticulture, Agawam, MA, USA) cold-stratified for 2–3 days in the dark at 6–8 °C, then grown in AR-66L growth chambers (Percival Scientific, Perry, IA, USA), equipped with both incandescent and fluorescent light bulbs, at an average light intensity of 175 μE/m^2^/s. Plants were grown under a 16 h light:8 h dark cycle at 22 °C. All transgenic lines were generated by floral dip [36] then selected on soil treated with basta herbicide. Segregating T_2_ seed samples harvested from T_1_ plants were sampled in bulk and analyzed for changes in lipid composition as described below.

### Fatty acid analysis by gas chromatography

Plant organ and yeast cellular lipid composition was determined by gas chromatography (GC) analysis of fatty acid methyl esters (FAME). Plant samples were mixed with sulfuric acid (5% vol/vol in dry methanol) and incubated at 80–90 °C for 1–2 h, in 100 mm glass GC tubes capped with teflon-lined caps. FAMEs were extracted with hexane and analyzed on an Agilent Technologies 6890N gas chromatograph with flame ionization detection (FID) using split injection on a SP-2380 column (Supelco, Bellefonte, PA, 60 m × 0.25 mm inner diameter, 0.2 µm thickness). Separation was achieved using a temperature program ramping from 170–215 ˚C at 5 ˚C/min, followed by a 6 min hold at 215 ˚C. Percentages of each FAME were calculated based on peak area counts. FAMEs were identified by co-migration with GC standards.

## Supplementary Information

**Additional file 1: Fig. S1.** Representative gas chromatography traces of yeast strain MMY011a, transformed with empty vector pESC-HIS (A), Gb713 FAD2-1A (B), mutant Gb713 fad2-1d (C) or repaired Gb713 FAD2-1D. See Figure 4 for full analysis. The primary fatty acids C16:0, C16:1, C18:0 and C18:1 elute at approximate retention times 10.03, 10.78, 12.52, and 13.17 minutes, respectively. The novel C16:2 product produced by FAD2-1A and FAD2-1D elutes at 11.99 minutes. The arrows indicate the FAD2 product C18:2 at 14.22 minutes, note the small amount of C18:2 produced by fad2-1d in (C). **Table S1.** Screenshot of a portion of the large nucleotide sequence identity matrix used to compare the numbers, identities, and chromosomal locations of the 284 copies and 124 copies of the retrotransposon found in *G. barbadense *and* G. hirsutum*, respectively, as described in the “Transposable element identification and molecular characterization” portion of the Results section. The full file is freely available upon request. **Table S2.** Synthetic Neon Green/FAD2/NeonGreen fusion expression sequences. NeonGreen protein sequence boxed in green. Start methionine ATG codon for NeonGreen was removed in AtFAD2 and Gb713 fad2-1d fusions, remainder of coding sequence was fused in-frame with 8-residue flexible linker (underlined).

## Data Availability

The nucleotide sequence of the retrotransposon here described has been deposited in Genbank as accession OP970913. Sharing of other raw data and materials described in this study will be considered for all reasonable requests, in accordance with USDA policies and procedures. Please contact Jay Shockey (Jay.Shockey@usda.gov) to request raw data or other materials.
