# Supplementary material for: A mutant cotton fatty acid desaturase 2-1d allele causes protein mistargeting and altered seed oil composition
Source: BMC Plant Biol. 2023 Mar 17;23:147. doi: 10.1186/s12870-023-04160-8 (PMC10021949; doi:10.1186/s12870-023-04160-8)
Supplement: Supplementary file 1 — Additional file 1: Fig. S1. Representative gas chromatography traces of yeast strain MMY011a, transformed with empty vector pESC-HIS (A), Gb713 FAD2-1A (B), mutant Gb713 fad2-1d (C) or repaired Gb713 FAD2-1D. See Figure 4 for full analysis. The primary fatty acids C16:0, C16:1, C18:0 and C18:1 elute at approximate retention times 10.03, 10.78, 12.52, and 13.17 minutes, respectively. The novel C16:2 product produced by FAD2-1A and FAD2-1D elutes at 11.99 minutes. The arrows indicate the FAD2 product C18:2 at 14.22 minutes, note the small amount of C18:2 produced by fad2-1d in (C). Table S1. Screenshot of a portion of the large nucleotide sequence identity matrix used to compare the numbers, identities, and chromosomal locations of the 284 copies and 124 copies of the retrotransposon found in G. barbadense and G. hirsutum, respectively, as described in the “Transposable element identification and molecular characterization” portion of the Results section. The full file is freely available upon request. Table S2. Synthetic Neon Green/FAD2/NeonGreen fusion expression sequences. NeonGreen protein sequence boxed in green. Start methionine ATG codon for NeonGreen was removed in AtFAD2 and Gb713 fad2-1d fusions, remainder of coding sequence was fused in-frame with 8-residue flexible linker (underlined). [file 12870_2023_4160_MOESM1_ESM.docx]

Fig. S1

Representative gas chromatography traces of yeast strain MMY011a, transformed with empty vector pESC-HIS (A), Gb713 FAD2-1A (B), mutant Gb713 fad2-1d (C) or repaired Gb713 FAD2-1D. See Figure 4 for full analysis. The primary fatty acids C16:0, C16:1, C18:0 and C18:1 elute at approximate retention times 10.03, 10.78, 12.52, and 13.17 minutes, respectively. The novel C16:2 product produced by FAD2-1A and FAD2-1D elutes at 11.99 minutes. The arrows indicate the FAD2 product C18:2 at 14.22 minutes, note the small amount of C18:2 produced by fad2-1d in (C).


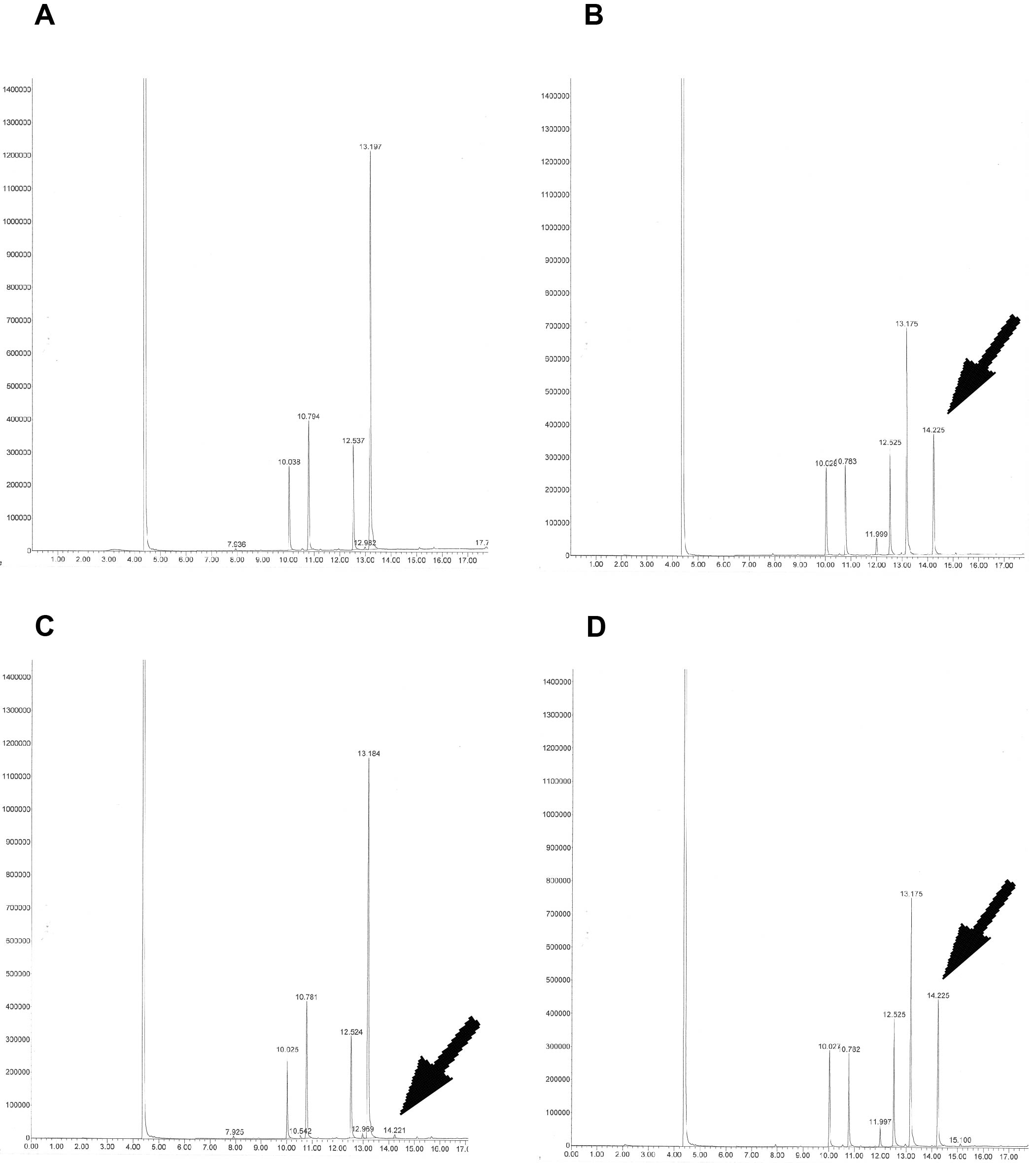


Table S1. Screenshot of a portion of the large nucleotide sequence identity matrix used to compare the numbers, identities, and chromosomal locations of the 284 copies and 124 copies of the retrotransposon found in *G. barbadense* and *G. hirsutum*, respectively, as described in the “Transposable element identification and molecular characterization” portion of the Results section. The full file is freely available upon request.


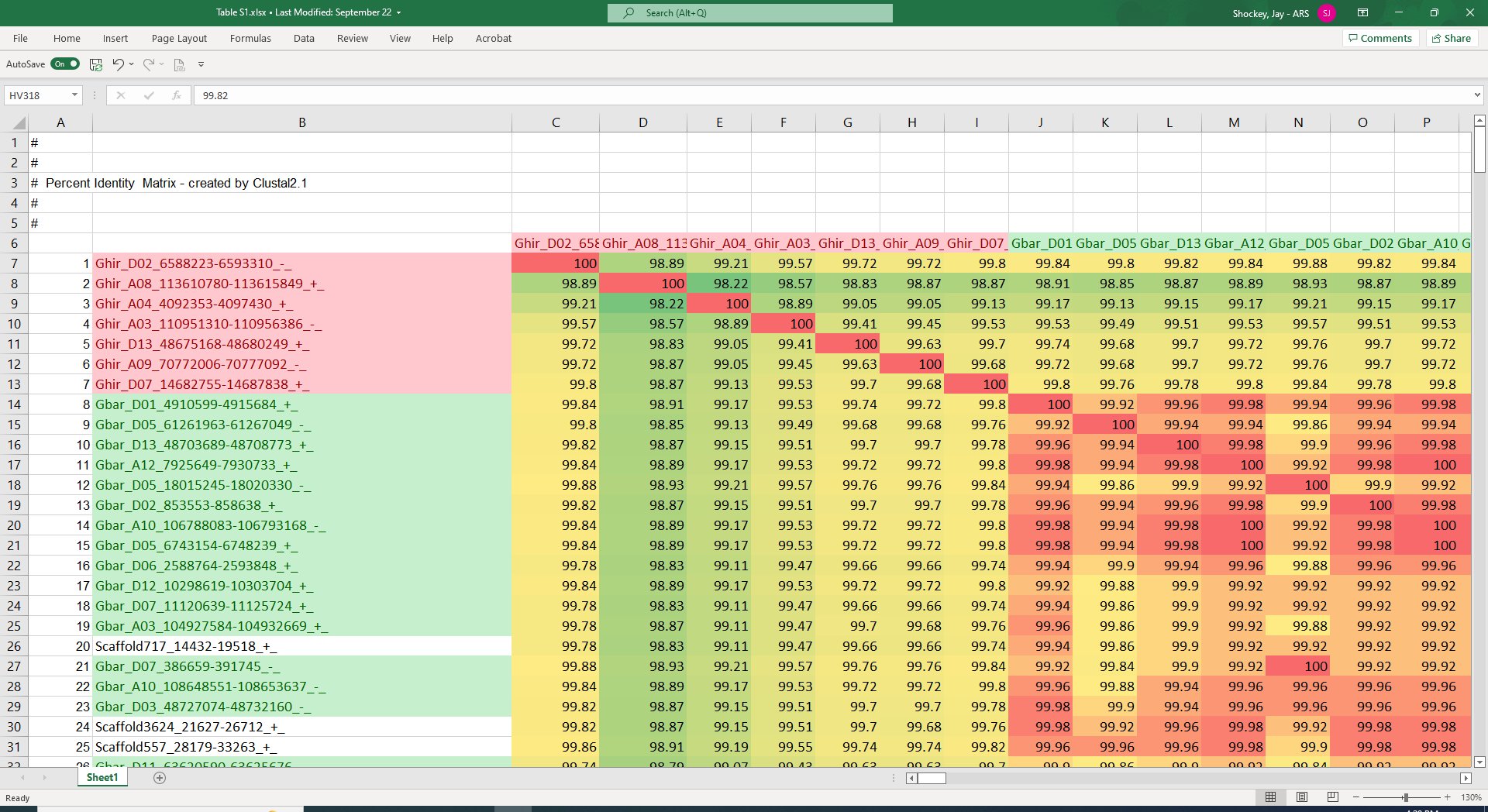


Table S2. Synthetic Neon Green/FAD2/NeonGreen fusion expression sequences. NeonGreen protein sequence boxed in green. Start methionine ATG codon for NeonGreen was removed in AtFAD2 and Gb713 fad2-1d fusions, remainder of coding sequence was fused in-frame with 8-residue flexible linker (underlined).

ATGGTTTCGAAAGGAGAGGAGGATAATATGGCTAGCCTCCCAGCGACCCACGAACTGCATATTTTTGGCAGCATTAATGGCGTTGACTTTGATATGGTGGGGCAGGGAACAGGGAACCCTAACGATGGCTATGAGGAGCTCAATCTCAAGAGTACAAAAGGAGATTTGCAATTTTCACCTTGGATCCTGGTTCCGCATATTGGCTACGGCTTTCATCAATACTTGCCTTATCCGGACGGCATGTCCCCGTTCCAAGCTGCGATGGTGGATGGTTCTGGATACCAGGTGCACCGTACTATGCAGTTTGAGGACGGTGCCTCACTGACGGTCAACTATAGATATACTTATGAAGGCTCACACATTAAGGGTGAGGCCCAAGTTAAAGGAACAGGGTTTCCTGCGGATGGACCGGTAATGACAAACAGTTTAACCGCTGCGGACTGGTGTCGCTCGAAAAAAACATACCCAAACGATAAAACGATCATCTCGACCTTCAAATGGAGCTATACTACGGGCAACGGCAAACGCTATCGTTCCACAGCACGCACGACTTATACGTTTGCTAAACCGATGGCCGCAAACTACCTCAAAAATCAACCTATGTACGTGTTCAGAAAAACCGAGTTAAAACATTCAAAAACGGAACTTAATTTTAAAGAGTGGCAAAAGGCGTTTACAGACGTGATGGGTATGGATGAACTCTATAAGTGA

MVSKGEEDNMASLPATHELHIFGSINGVDFDMVGQGTGNPNDGYEELNLKSTKGDLQFSPWILVPHIGYGFHQYLPYPDGMSPFQAAMVDGSGYQVHRTMQFEDGASLTVNYRYTYEGSHIKGEAQVKGTGFPADGPVMTNSLTAADWCRSKKTYPNDKTIISTFKWSYTTGNGKRYRSTARTTYTFAKPMAANYLKNQPMYVFRKTELKHSKTELNFKEWQKAFTDVMGMDELYK*

AtFAD2-cNeonGreen

ATGGGTGCAGGTGGAAGAATGCCGGTTCCTACTTCTTCCAAGAAATCGGAAACCGACACCACAAAGCGTGTGCCGTGCGAGAAACCGCCTTTCTCGGTGGGAGATCTGAAGAAAGCAATCCCGCCGCATTGTTTCAAACGCTCAATCCCTCGCTCTTTCTCCTACCTTATCAGTGACATCATTATAGCCTCATGCTTCTACTACGTCGCCACCAATTACTTCTCTCTCCTCCCTCAGCCTCTCTCTTACTTGGCTTGGCCACTCTATTGGGCCTGTCAAGGCTGTGTCCTAACTGGTATCTGGGTCATAGCCCACGAATGCGGTCACCACGCATTCAGCGACTACCAATGGCTGGATGACACAGTTGGTCTTATCTTCCATTCCTTCCTCCTCGTCCCTTACTTCTCCTGGAAGTATAGTCATCGCCGTCACCATTCCAACACTGGATCCCTCGAAAGAGATGAAGTATTTGTCCCAAAGCAGAAATCAGCAATCAAGTGGTACGGGAAATACCTCAACAACCCTCTTGGACGCATCATGATGTTAACCGTCCAGTTTGTCCTCGGGTGGCCCTTGTACTTAGCCTTTAACGTCTCTGGCAGACCGTATGACGGGTTCGCTTGCCATTTCTTCCCCAACGCTCCCATCTACAATGACCGAGAACGCCTCCAGATATACCTCTCTGATGCGGGTATTCTAGCCGTCTGTTTTGGTCTTTACCGTTACGCTGCTGCACAAGGGATGGCCTCGATGATCTGCCTCTACGGAGTACCGCTTCTGATAGTGAATGCGTTCCTCGTCTTGATCACTTACTTGCAGCACACTCATCCCTCGTTGCCTCACTACGATTCATCAGAGTGGGACTGGCTCAGGGGAGCTTTGGCTACCGTAGACAGAGACTACGGAATCTTGAACAAGGTGTTCCACAACATTACAGACACACACGTGGCTCATCACCTGTTCTCGACAATGCCGCATTATAACGCAATGGAAGCTACAAAGGCGATAAAGCCAATTCTGGGAGACTATTACCAGTTCGATGGAACACCGTGGTATGTAGCGATGTATAGGGAGGCAAAGGAGTGTATCTATGTAGAACCGGACGGAGGTTCAGGAGGTTCTGGCAGTGTTTCGAAAGGAGAGGAGGATAATATGGCTAGCCTCCCAGCGACCCACGAACTGCATATTTTTGGCAGCATTAATGGCGTTGACTTTGATATGGTGGGGCAGGGAACAGGGAACCCTAACGATGGCTATGAGGAGCTCAATCTCAAGAGTACAAAAGGAGATTTGCAATTTTCACCTTGGATCCTGGTTCCGCATATTGGCTACGGCTTTCATCAATACTTGCCTTATCCGGACGGCATGTCCCCGTTCCAAGCTGCGATGGTGGATGGTTCTGGATACCAGGTGCACCGTACTATGCAGTTTGAGGACGGTGCCTCACTGACGGTCAACTATAGATATACTTATGAAGGCTCACACATTAAGGGTGAGGCCCAAGTTAAAGGAACAGGGTTTCCTGCGGATGGACCGGTAATGACAAACAGTTTAACCGCTGCGGACTGGTGTCGCTCGAAAAAAACATACCCAAACGATAAAACGATCATCTCGACCTTCAAATGGAGCTATACTACGGGCAACGGCAAACGCTATCGTTCCACAGCACGCACGACTTATACGTTTGCTAAACCGATGGCCGCAAACTACCTCAAAAATCAACCTATGTACGTGTTCAGAAAAACCGAGTTAAAACATTCAAAAACGGAACTTAATTTTAAAGAGTGGCAAAAGGCGTTTACAGACGTGATGGGTATGGATGAACTCTATAAGAGGGAAGGTGACAAGAAAGGTGTGTACTGGTACAACAATAAGTTATGA

MGAGGRMPVPTSSKKSETDTTKRVPCEKPPFSVGDLKKAIPPHCFKRSIPRSFSYLISDIIIASCFYYVATNYFSLLPQPLSYLAWPLYWACQGCVLTGIWVIAHECGHHAFSDYQWLDDTVGLIFHSFLLVPYFSWKYSHRRHHSNTGSLERDEVFVPKQKSAIKWYGKYLNNPLGRIMMLTVQFVLGWPLYLAFNVSGRPYDGFACHFFPNAPIYNDRERLQIYLSDAGILAVCFGLYRYAAAQGMASMICLYGVPLLIVNAFLVLITYLQHTHPSLPHYDSSEWDWLRGALATVDRDYGILNKVFHNITDTHVAHHLFSTMPHYNAMEATKAIKPILGDYYQFDGTPWYVAMYREAKECIYVEPDGGSGGSGSVSKGEEDNMASLPATHELHIFGSINGVDFDMVGQGTGNPNDGYEELNLKSTKGDLQFSPWILVPHIGYGFHQYLPYPDGMSPFQAAMVDGSGYQVHRTMQFEDGASLTVNYRYTYEGSHIKGEAQVKGTGFPADGPVMTNSLTAADWCRSKKTYPNDKTIISTFKWSYTTGNGKRYRSTARTTYTFAKPMAANYLKNQPMYVFRKTELKHSKTELNFKEWQKAFTDVMGMDELYKREGDKKGVYWYNNKL*

Gb713-21dtrunc-cNeonGreen

ATGGGTGCCGGTGGTAGGATGCCAATTGACGGTATAAAGGAGGAAAATCGAGGCTCGGTCAATCGAGTTCCGATCGAGAAGCCTCCGTTTACGCTCGGTCAGATCAAGCAAGCCATTCCGCCCCACTGTTTTCGCCGCTCCCTCCTTCGATCCTTCTCCTACGTGGTCCATGACCTATGCTTAGCCTCTCTCTTTTACTACATTGCAACATCATATTTTCACTTTCTCCCACAACCCTTTTCCTACATTGCTTGGCCTGTCTATTGGGTTCTCCAAGGTTGCATCCTCACCGGTGTTTGGGTCATCGCACACGAATGCGGTCACCACGCTTTCAGTGACTACCAATGGGTTGACGACACCGTCGGGTTGATCCTTCACTCCGCCCTTTTAGTCCCGTACTTCTCGTGGAAAATCAGTCACCGCCGTCACCACTCGAACACCGGTTCCATGGAGCGTGACGAAGTATTCGTGCCCAAACCCAAGTCTAAATTATCATGCTTTGCGAAATACTTCAACAATCCACCCGGTCGAGTTCTCTCTCTTGTAGTCACATTGACTCTTGGTTGGCATATGTACTTAGCCTTCAACGTTTCGGGTCGATACTATGATCGATTAGCTTCCCACTATAACCCTTACGGCCCCATTTACTCCGAACGCGAGAGGCTACAAGTTTACATCTCCGATGCTGGTATAGTTGCGGTAATTTATGTACTTTATAAGATTGCTGCAACAAAAGGGCTGGCTTGGCTTTTATGCACTTATGGGGTACCTCTACTTATTGTGAATGCCTTCCTTGTGTTGATCACCTACTTGCAACATACTCACTCGGCATTGCCGCATTACGACTCGTCTGAATGGGATTGGTTTCGAGGAGCATTGTCGACGATTGATCGAGATTACGGGGTGTTGAACAAAGTGTTCCATAACATCACCGATACGCATGTGGCTCATCACCTCTTCTCAACGATGCCACATTATCATGCAATGGAGGCCACTAAAGCAATCAAACCGATACTCGGCAAGTATTATCCTTTCGACGGGACACCGATTTATAAGGCAATGTGGAGGGAGGCAAAAGAGTGCCTTTACGTCGAGGCTGACGGAGGTTCAGGAGGTTCTGGCAGTGTTTCGAAAGGAGAGGAGGATAATATGGCTAGCCTCCCAGCGACCCACGAACTGCATATTTTTGGCAGCATTAATGGCGTTGACTTTGATATGGTGGGGCAGGGAACAGGGAACCCTAACGATGGCTATGAGGAGCTCAATCTCAAGAGTACAAAAGGAGATTTGCAATTTTCACCTTGGATCCTGGTTCCGCATATTGGCTACGGCTTTCATCAATACTTGCCTTATCCGGACGGCATGTCCCCGTTCCAAGCTGCGATGGTGGATGGTTCTGGATACCAGGTGCACCGTACTATGCAGTTTGAGGACGGTGCCTCACTGACGGTCAACTATAGATATACTTATGAAGGCTCACACATTAAGGGTGAGGCCCAAGTTAAAGGAACAGGGTTTCCTGCGGATGGACCGGTAATGACAAACAGTTTAACCGCTGCGGACTGGTGTCGCTCGAAAAAAACATACCCAAACGATAAAACGATCATCTCGACCTTCAAATGGAGCTATACTACGGGCAACGGCAAACGCTATCGTTCCACAGCACGCACGACTTATACGTTTGCTAAACCGATGGCCGCAAACTACCTCAAAAATCAACCTATGTACGTGTTCAGAAAAACCGAGTTAAAACATTCAAAAACGGAACTTAATTTTAAAGAGTGGCAAAAGGCGTTTACAGACGTGATGGGTATGGATGAACTCTATAAGGTTGGTGGTGGTGGTAGCTGTTAA

MGAGGRMPIDGIKEENRGSVNRVPIEKPPFTLGQIKQAIPPHCFRRSLLRSFSYVVHDLCLASLFYYIATSYFHFLPQPFSYIAWPVYWVLQGCILTGVWVIAHECGHHAFSDYQWVDDTVGLILHSALLVPYFSWKISHRRHHSNTGSMERDEVFVPKPKSKLSCFAKYFNNPPGRVLSLVVTLTLGWHMYLAFNVSGRYYDRLASHYNPYGPIYSERERLQVYISDAGIVAVIYVLYKIAATKGLAWLLCTYGVPLLIVNAFLVLITYLQHTHSALPHYDSSEWDWFRGALSTIDRDYGVLNKVFHNITDTHVAHHLFSTMPHYHAMEATKAIKPILGKYYPFDGTPIYKAMWREAKECLYVEADGGSGGSGSVSKGEEDNMASLPATHELHIFGSINGVDFDMVGQGTGNPNDGYEELNLKSTKGDLQFSPWILVPHIGYGFHQYLPYPDGMSPFQAAMVDGSGYQVHRTMQFEDGASLTVNYRYTYEGSHIKGEAQVKGTGFPADGPVMTNSLTAADWCRSKKTYPNDKTIISTFKWSYTTGNGKRYRSTARTTYTFAKPMAANYLKNQPMYVFRKTELKHSKTELNFKEWQKAFTDVMGMDELYKVGGGGSC*

TruncEH-repQCt: CTATAAGGTTGGTGGTGGTGGTGGTGGTAGCAAAGGTGTTTTTTGGTATCGTAACAAGTTCTGATTAATTAACAATTCTTCGC

TruncEH-repQCr GCGAAGAATTGTTAATTAATCAGAACTTGTTACGATACCAAAAAACACCTTTGCTACCACCACCACCACCACCAACCTTATAG

-primer sequences were used to ‘repair’ truncated Gb713 fad2-1d 3’ coding sequence (Figure 1).
